# Sound Localization in Real-Time Vocoded Cochlear-Implant Simulations With Normal-Hearing Listeners

**DOI:** 10.1177/2331216519847332

**Published:** 2019-05-14

**Authors:** Sebastian A. Ausili, Bradford Backus, Martijn J. H. Agterberg, A. John van Opstal, Marc M. van Wanrooij

**Affiliations:** 1 Department of Biophysics, Donders Institute for Brain, Cognition and Behaviour, Radboud University, Nijmegen, the Netherlands; 2 Oticon Medical, Copenhagen, Denmark; 3 Department of Otorhinolaryngology, Donders Institute for Brain, Cognition and Behaviour, Radboud University Nijmegen Medical Center, the Netherlands

**Keywords:** cochlear implant, auditory perception, psychoacoustics, acoustic stimulation

## Abstract

Bilateral cochlear-implant (CI) users and single-sided deaf listeners with a CI are less effective at localizing sounds than normal-hearing (NH) listeners. This performance gap is due to the degradation of binaural and monaural sound localization cues, caused by a combination of device-related and patient-related issues. In this study, we targeted the device-related issues by measuring sound localization performance of 11 NH listeners, listening to free-field stimuli processed by a real-time CI vocoder. The use of a real-time vocoder is a new approach, which enables testing in a free-field environment. For the NH listening condition, all listeners accurately and precisely localized sounds according to a linear stimulus–response relationship with an optimal gain and a minimal bias both in the azimuth and in the elevation directions. In contrast, when listening with bilateral real-time vocoders, listeners tended to orient either to the left or to the right in azimuth and were unable to determine sound source elevation. When listening with an NH ear and a unilateral vocoder, localization was impoverished on the vocoder side but improved toward the NH side. Localization performance was also reflected by systematic variations in reaction times across listening conditions. We conclude that perturbation of interaural temporal cues, reduction of interaural level cues, and removal of spectral pinna cues by the vocoder impairs sound localization. Listeners seem to ignore cues that were made unreliable by the vocoder, leading to acute reweighting of available localization cues. We discuss how current CI processors prevent CI users from localizing sounds in everyday environments.

## Introduction

Cochlear implants (CIs) are increasingly used to restore severe-to-profound hearing loss. Nowadays, CI recipients can achieve high levels of speech understanding, which greatly improves their quality of life, their ability to engage in social interactions, and their cognitive and linguistic development (Capretta & Moberly, 2015; Schorr, Roth, & Fox, 2009; Sladen & Zappler, 2015; Vermeire et al., 2005). Because of this success, the indication criteria for CI implantation have been extended to include a wider variety of hearing loss and pathologies. So far, the only way to restore bilateral input in the profoundly deaf auditory system is bilateral CI implantation. Similarly, single-sided deafness can be overcome by CI implantation in the deaf ear (i.e., stimulation of the deprived auditory pathway). As a result of restored bilateral input, spatial hearing abilities can in principle be improved (Bernstein, Goupell, Schuchman, Rivera, & Brungart, 2016; Mertens, De Bodt, &Van De Heyning, 2017; Zeitler et al., 2015).

However, CI users are still poor at localizing sounds (Grantham, Ashmead, Ricketts, Labadie, & Haynes, 2007; Jones, Kan, & Litovsky, 2014; Nopp, Schleich, & D’Haese, 2004), especially when compared with normal-hearing (NH) listeners. This poor performance is due to the degradation of sound localization cues by a combination of device-related and patient-related issues (Gifford et al., 2013; Vinay & Moore, 2007; O’Connell, Dedmon, & Haynes, 2017). For example, a patient-related issue is the degradation of auditory neural pathways that are affected by the patient’s hearing loss. Instead, a device-related issue is associated with the CI device itself, which, after processing the acoustic input, could lead to inconsistent and ambiguous sound localization cues (Fitzgerald, Kan, & Goupell, 2015; Kelvasa & Dietz, 2015).

For NH listeners, the localization cues in the horizontal plane (azimuth) consist primarily of interaural level differences (ILDs) and interaural time differences (ITDs) for high-frequency (>3000 Hz) and low-frequency (<1500 Hz) sounds, respectively (Blauert, 1996; Rayleigh, 1907). High-frequency (>4000 Hz) monaural spectral-shape cues that arise from the sound wave interactions with the head and pinnae enable localization in the median plane (elevation; Hofman, Van Riswick, & Van Opstal, 1998; Otte, Agterberg, Van Wanrooij, Snik, & Van Opstal, 2013; Van Wanrooij & Van Opstal, 2005; Wightman & Kistler, 1989).

The majority of the current CI systems stimulate the auditory nerve with a constant rate of electrical pulses that is modulated by the temporal envelope of the sound wave. As a result of this encoding strategy, the high-frequency temporal fine structure of the sound is not provided to the auditory system (Wilson & Dorman, 2008). In addition, current bilateral systems do not apply interaural device synchronization, yielding independent, uncorrelated fine-structure signals for both ears. In this way, the ITDs could be severely distorted. Moreover, having the microphones outside the pinnae severely affects the frequency-dependent ILDs as well as the subtle monaural spectral pinna cues (Jones, Kan, & Litovsky, 2016).

The aim of this study was to determine—through real-time vocoder simulation—how sound localization in NH listeners is affected (acutely) by these device-related issues. To that end, we tested horizontal and vertical sound localization performance of NH listeners in a free-field environment for three listening conditions: two NH ears, bilateral CI vocoders, and a unilateral CI vocoder with an NH ear.

We applied the Oticon Medical (OM) Research Platform, provided by Oticon Medical (Backus, Adiloğlu, & Herzke, 2015), as a real-time vocoder, in which the sound is recorded and processed online. This novel technique enabled testing in free-field listening conditions, rather than with virtual acoustic stimulation (Goupell, Majdak, & Laback, 2010; Jones et al., 2014). The latter typically leads to poorer performance than free-field localization (Wenzel, Arruda, Kistler, & Wightman, 1993) and often requires training and explicit feedback (Majdak, Goupell, & Laback, 2010, 2011; Wenzel et al., 1993).

While vocoders are useful to evaluate device-related issues in speech perception (e.g., Dorman & Loizou, 1997; Dorman, Loizou, & Fitzke, 1998; Friesen, Shannon, Baskent, & Wang, 2001; Fu & Nogaki, 2005; Garadat, Litovsky, Yu, & Zeng, 2009; Goupell et al., 2010; Goupell, Stoelb, Kan, & Litovsky, 2018; Litvak, Spahr, Saoji, & Fridman, 2007; Qin & Oxenham, 2003; Shannon, Fu, & Galvin Iii, 2004; Shannon, Zeng, Kamath, Wygonski, Ekelid, 1995; Whitmal, Poissant, Freyman, & Helfer, 2007; Wilson et al., 1991), to our knowledge, only three vocoded CI simulation studies have so far addressed sound localization with off-line processing. Seeber and Hafter (2011) reported the absence of a precedence effect in NH listeners with a bilateral noise-band vocoder, as listeners persistently perceived two auditory images (one leading and one lagging source). Goupell et al. (2010) showed that localization performance on the medial plane was worse for spectrally degraded vocoded stimuli. These authors also mentioned and discussed the drawbacks of localizing virtual versus real sound sources. Jones et al. (2014) found impoverished virtual horizontal sound localization in NH listeners with bilateral vocoders, which was comparable to the response patterns of bilateral CI users (depending on the carrier used). With our novel experimental paradigm, we expand on these studies by including a unilateral (with an NH ear on the contralateral side) CI vocoder simulation. This will allow for an examination of the role of monaural spectral cues and the effect of potentially incongruent inputs on sound azimuth and elevation localization performance simultaneously. Moreover, the technique allows one to determine how the binaural localization cues are reweighted by analyzing localization performance as a function of sound source azimuth.

## Methods

### Listeners

Eleven NH listeners (aged 25–33 years; seven men) participated in the experiments. All had NH (within 20 dB of audiometric zero) as determined by a standard pure-tone audiogram (ISO 8253-1:2010). None of the listeners had any uncorrected visual disorder.

Although four listeners were experienced with the experimental methodology carried out in the laboratory, all listeners were naive as to the purpose of the study, except for two authors. All experimental procedures have been approved by the local ethics committee of the Faculty of Social Sciences of the Radboud University (ECSW 2016-2208-41), as they concerned noninvasive observational experiments with healthy adult human listeners. Prior to their participation in the experiments, volunteers gave their written informed consent.

### Vocoder

**Figure 1.**
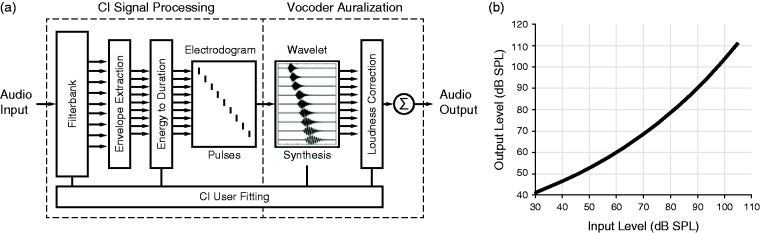
(a) Diagram showing the processing blocks of one side of the CI simulation vocoder. (b) Input versus output acoustic levels for the 1 kHz electrode. Other electrodes differed slightly due to the frequency dependency contained in the ISO 226 standard. CI = cochlear implant; dB SPL = decibel of sound pressure level.

A real-time bilateral vocoder was used to simulate the processing of a bilateral CI device. The vocoder was provided by Oticon Medical as part of the Oticon Medical Research Platform and is based on the previous work (Bräcker, Hohmann, Kollmeier, & Schulte, 2009; Langner & Jürgens, 2016). It operates with fixed, identical latencies of ∼7 ms on each side, which is in the range of existing clinical CI systems (Wess, Brungart, & Bernstein, 2017; Zirn, Arndt, & Wesarg, 2015). While this is a considerable latency, this delay is below the echo threshold (Brown, Stecker, & Tollin, 2015); listeners did not report perceiving two sounds in the listening condition where sounds are heard though an NH ear and through a vocoder. The signal processing flow is shown in Figure 1(a).

Two microphone signals (front-facing omnidirectional microphones) were acquired from calibrated behind-the-ear microphones (BTEs), which had a flat frequency response over the acoustic range of interest (±1 dB, 0.1–10 kHz). Both inputs were digitized using the same 16.667 kHz sample clock. Signals were subsequently passed through the basic OM Saphyr CI processing algorithm, which included (a) an fast-fourier-transform-based filter bank, (b) an envelope energy extraction stage, and (c) an energy to biphasic pulse-duration mapping stage. No compression was used except for an instantaneous hard output limit at 105 decibel of sound pressure level (dB SPL). This CI processing scheme is equivalent to a 20-channel continuous interleaved sampling strategy (Wilson, Finley, Lawson, Wolford, & Zerbi, 1993) with an electrode interpulse rate of ∼520 Hz and sound intensity encoded by pulse duration. The default OM Saphyr clinical fitting (Table 1) was used for the left and right ears. Using this processing, a pair of binaural electrodograms was first formed.

**Table 1.** Electrode Fitting Parameters From the Saphyr Demo Fitting File Showing the Electrode Numbers and Their Associated Center Frequencies, Frequency Bands, BWs, and Threshold “T and C-Level” Durations.

*E*#	*f*_cf_ (Hz)	*f*_low_ (Hz)	*f*_high_ (Hz)	BW (Hz)	*d_t_* (µs)	*d_c_* (µs)
20	260	195	326	131	28	48
19	380	326	456	130	33	56
18	520	456	586	130	36	64
17	651	586	716	130	38	68
16	781	716	846	130	37	70
15	911	846	977	131	38	72
14	1042	977	1107	130	38	74
13	1172	1107	1237	130	37	74
12	1302	1237	1367	130	39	75
11	1432	1367	1497	130	39	74
10	1627	1497	1758	260	41	75
9	1888	1758	2018	260	41	70
8	2213	2018	2409	391	40	69
7	2604	2409	2799	390	39	67
6	3125	2799	3451	652	37	64
5	3776	3451	4102	651	35	63
4	4492	4102	4883	781	30	60.5
3	5338	4883	5794	911	27	57
2	6315	5794	6836	1042	25	49.5
1	7422	6836	8008	1172	23.5	39.5

*Note.* These same parameters were used for both the cochlear implant processing and wavelet synthesis stages of the vocoder. BW = bandwidth.

The wavelet vocoder was built to take binaural electrodograms as input. This approach allowed us to include all information contained within the electrodograms as part of the input, but it also allowed the vocoder to reproduce the 520 Hz fixed-pulse rate as a pitch percept at its output—something CI users do not hear. To reduce the stimulation pitch percept, a random jitter (mean = 10%) was artificially introduced to both electrodograms prior to auralization. This reduced the stimulation pitch to a level that was not noticed by the listeners.

The vocoder processed the adjusted incoming left and right electrodograms back into a stereo audio signal by using the CI-fitting data of Table 1 as parameter inputs in the following way:

Each electrode pulse produced a third-order gammatone wavelet according to:

(1) $$\begin{array}{c} g(t)={\mathrm{at}}^{2}e^{-2\pi \mathrm{bt}}\text{cos}(2\pi \mathrm{ft}+Ø){|}_{t\geq 0} \end{array}$$

consisting of a constant factor *a*, an envelope $v(t)=e^{-2\pi \mathrm{bt}}$, and a fine structure cos(2πft+Ø). The parameters *f* and *b* were calculated directly from the CI fitting. The carrier *f* was set to the center frequency of the electrode band. The damping factor *b* was calculated as b=B/π, with *B* the electrode bandwidth. No attempt was made to include the phenomenon of current spread within this bandwidth.

Parameter *a* was calculated from the duration of the pulse in the electrogram. The phase parameter ∅ was set such that a maximum in the carrier coincided with the envelope maximum. The amplitude parameter *a* was tuned to create the appropriate root-mean-square (RMS) acoustic energy within the band, according to the input–output function as shown in Figure 1(b). In cases where the stimulation rate was high compared with the bandwidth, and wavelets overlapped to produce constructive or destructive interference, *a* and ∅ were adjusted together to ensure that the RMS energy was preserved within each 1.91-ms analysis time slice.

Each wavelet’s amplitude was fully determined by the duration of the electrode’s pulse in relation to the *T* and *C* durations given in Table 1. The mapping between the electrode’s pulse duration, *d_e_*, and the vocoder loudness (*L*_phons_, in phons) was specified by

(2) $$\begin{array}{c} d_{e}=d_{t}\cdot (\frac{L_{\text{phons}}}{32}+1{)}^{0.8333\cdot {\text{log}}_{e}(\frac{d_{c}}{d_{t}})} \end{array}$$

with *d_t_* the threshold duration and *d_c_* the duration of the maximum comfortable level (or C-level), both taken from Table 1.

The RMS acoustic energy was calculated from *L*_phons_ back to dB SPL in each band by using the conversion of the ISO-226 standard, which was linearly extrapolated above 90 phons. The mapping gives a natural output range from 0 to 74 phons in response to an input dynamic range from 30 to 105 dB SPL.

The output range was shifted using a headphone volume control to equate input and output levels at 60 dB(A) (measured in dB SPL on A-weighted scale) using calibrated broadband (BB) noise as input. An example of the whole system input versus output level function is shown (Figure 1(b)).

**Figure 2.**
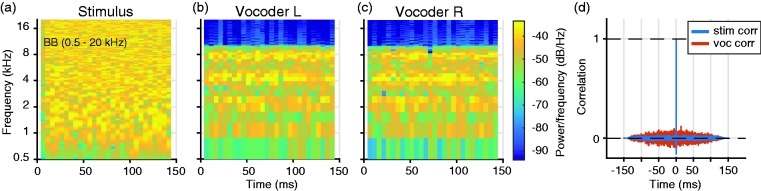
Spectrograms of a BB stimulus and vocoder output. The acoustic stimulus (a) is processed by the vocoder and generated the left (b) and right (c) output (limited between 0.2 and 8 kHz). The cross-correlation of the acoustic stimulus (in blue) shows a peak at 0 ms (d), which will shift according to the applied ITD. The correlation between the right and left output (in red) shows no correlation between each other. BB = broadband.

The final output was determined by the sum of the wavelets (Figure 2). All interpulse periods were pseudo-constant (∼520 Hz) and were not synchronized between the right and left devices. As a result, the temporal fine structure ITD was not preserved at the vocoder outputs (Figure 2(d)). Sound levels were measured in dB(A) with a Brüel & Kjaer 2236 (Nærum, Denmark). The acoustic output of the system was calibrated with an ear simulator (Brüel & Kjaer Type 4152, Nærum, Denmark).

### Localization Setup

The sound localization experiments were performed in a set-up that has been described in detail (Van Bentum, Van Opstal, Van Aartrijk, & Van Wanrooij, 2017). Briefly, the experiment took place in a dark, sound-attenuated room of 3.6 × 3.0 × 3.0 m with a background noise level below 20 dB(A). During the task, the listener sat comfortably in a chair in the center of a spherical wire frame, on which 125 small broad-range loudspeakers (SC 5.9; Visaton; art. no. 8006, Visaton GmbH & Co KG, Haan, Germany) had been mounted to cover the entire two-dimensional frontal field. Light-emitting diodes (LEDs; BIVAR Inc., Irvine, CA) were mounted at the center of each speaker and served as visual fixation stimuli for the head-movement calibration.

Head movements (Veugen, Hendrikse, et al., 2016) were recorded with the magnetic search coil technique (Robinson, 1963). To that end, a small coil was attached to the nose bridge of a lightweight spectacle frame. Along the edges of the room, three perpendicular pairs of coils generated the high-frequency oscillating magnetic fields that are needed to record the search coil’s orientation in all directions.

Sound playback and data acquisition were implemented by TDT3 data acquisition and stimulus generation hardware (Tucker-Davis Technologies, Alachua, FL), controlled by custom-made software written in MATLAB (version R2015a; The MathWorks Inc., Natick, MA).

### Stimuli

All sounds consisted of 150-ms Gaussian white noise bursts, with 5-ms sine-squared onset and offset ramps, that were BB (500 Hz to 20 kHz), low-pass (LP; 0.5–1.5 kHz), or high-pass (HP; 3.0–20 kHz) filtered. The noise bursts are well-localizable stimuli in the azimuth and elevation directions for NH listeners (Frens & Van Opstal, 1995; Goossens & Van Opstal, 1999; Middlebrooks & Green, 1991; Van Grootel, Van Wanrooij, & Van Opstal, 2011). In the vocoder simulation paradigms, the sounds were passed through the vocoder device according to the procedures described earlier.

**Figure 3.**
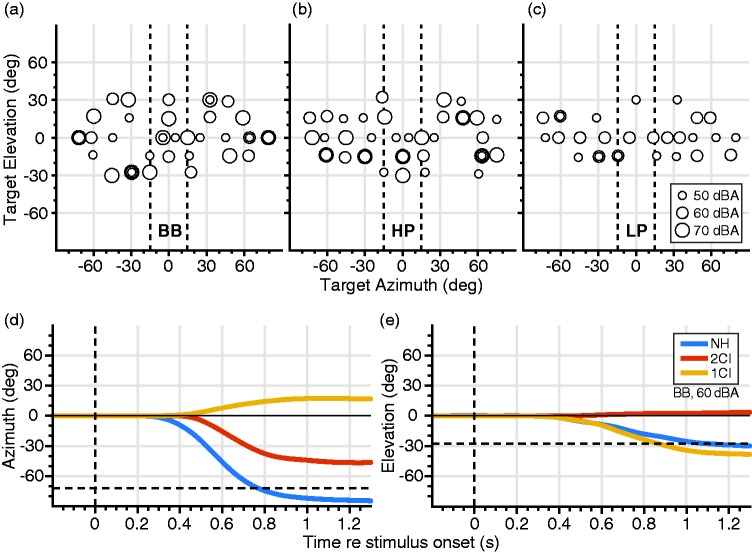
Localization paradigm. Target locations for (a) BB, (b) HP, and (c) LP filtered sounds for each sound level (represented by marker size). Vertical dashed lines indicate the ±15° boundaries of the central azimuth range. Head trajectories of example trials of listener S1 responding to a BB, 60 dB(A) sound in (d) azimuth (−72°), and (e) elevation (−28°), for the three listening conditions: normal hearing (NH—blue), bilateral CI vocoder (2CI—red), and unilateral CI vocoder with a contralateral NH ear (1CI—yellow). Horizontal dashed lines indicate target location; vertical dashed lines indicate target onset. BB = broadband; HP = high-pass; LP = low-pass.

BB and HP sounds were presented at 50, 60, and 70 dB(A), and LP sounds were presented at 50 and 60 dB(A). Fifteen different locations were presented per level. Sound locations were selected pseudo-randomly between ±70° in azimuth (α) and ±30° in elevation (ɛ) as indicated in Figure 3(a) to (c).

### Paradigms

#### Coil calibration

Prior to the actual experiments, we performed a visual coil calibration experiment. The listener had to accurately point with the head–fixed laser pointer of the spectacle frame to 24 known LED positions mounted on the spherical frame.

Stimulus and response coordinates were defined in double-pole azimuth–elevation reference frame (Knudsen & Konishi, 1979). A positive azimuth/elevation angle refers to targets and responses located on the right-hand/upward direction, with respect to straight ahead. The 24 fixation points obtained from the calibration experiment were used to train 2 three-layer back-propagation neural networks that served to calibrate the head-movement data in azimuth and elevation, respectively, and corrected any nonlinear inhomogeneities in the magnetic fields and cross-talk between the horizontal and vertical signals (Bremen et al., 2010; Goosens & Van Opstal, 1997; Van Barneveld & Van Wanrooij, 2013; Van Grootel et al., 2011). Both networks received the raw horizontal and vertical head-position signals (in V) as inputs and yielded the desired azimuth and elevation angles (in degrees) as their output with a precision of one degree, or better, over the entire measurement range.

#### Practice session

At the start of an experimental session, listeners were first familiarized with the experimental procedures in a short practice session of up to 15 trials. Listeners were instructed to orient their head (i.e., the laser pointer attached to the spectacle frame) as quickly and as accurately as possible to the perceived sound location. No feedback was given about actual localization performance.

#### Sound localization experiment

Every trial started with the presentation of a fixation LED at straight ahead, [α,ɛ] = [0°,0°]. Listeners had to point to this LED with the laser on the spectacle frame to ensure that the starting position of the head was always in the same straight-ahead direction in each trial. They then pressed a button, after which the fixation LED was turned off within 100 to 300 ms, and the sound stimulus was presented 200 ms later. The listener had to orient the head–fixed laser dot as fast and as accurately as possible to the perceived sound location. Head movements were recorded in azimuth and elevation for 1,500 ms (Figure 3(d) and (e)), after which the central fixation LED was switched on to start the next trial.

#### Listening conditions

Sound localization experiments were conducted for three listening conditions: (a) NH, (b) a bilateral vocoder (2CI), and (c) a unilateral vocoder in one ear, and NH in the contralateral ear (1CI). All listeners participated in the NH and 2CI listening conditions. Seven of the 11 listeners also participated in the 1CI condition, with 3(4) listeners having the vocoder on the right (left) hearing side.

### Data Analysis

#### Head-movement detection

Head movements were detected automatically from the calibrated head-position signals using a custom-made Matlab script that checked for head velocities exceeding 20° s^−1^. Onset and offset detection markings from the program were visually checked off-line and corrected when deemed necessary.

#### Regression analysis

Due to the nonlinear stimulus–response relationships in the 2CI and 1CI hearing conditions, a simple linear regression analysis across all conditions is not appropriate. We therefore divided the end points of the responses in all conditions into three nonoverlapping azimuth windows: left (−90° < α < −15°), right (+15° < α < +90°), and center (−15° ≤ α ≤ +15°; see Figure 3(a) to (c)). In each of these target ranges, we performed a linear regression (applying the least-squared error criterion) through the selected data points as follows:

(3) $$\alpha_{\text{R}}=\beta +\gamma \cdot \alpha_{\text{T}}$$

with $\alpha_{\text{T}}$ the target azimuth (in degrees), $\alpha_{\text{R}}$ the response azimuth (degrees), γ the slope (or: gain) of the best-fit regression line (dimensionless), and β the intercept (or: bias, in degrees). A gain close to 1 indicates accurate localization responses, while a gain close to 0 indicates the lack of a significant linear relationship. A perfect localization response would result in a gain of γ = 1 and a bias of β = 0°, and all three regression windows would yield the same result. A response pattern where the listener consistently directs the head to fixed locations in either the left or right hemisphere will yield gains close to 0 and large negative and positive bias values, respectively. Response variability, σ, was quantified by the standard deviation of the residuals around the best-fit line.

Localization performance in the elevation direction was analyzed by determining the regression line for the entire elevation range (−30° < ɛ < 30°), within each of nine nonoverlapping, contiguous 15°-wide windows in azimuth.

#### Promptness

Reaction times (in milliseconds) were measured by taking the difference between head-saccade onset and sound onset. Typically, their values followed a positively skewed distribution with an extended tail toward longer reaction times. For quantitative analysis across listeners, the reaction-time data were first transformed into their reciprocals (1/reaction time), in s^−1^, also known as promptness, which has been shown to follow a nearly Gaussian distribution (Carpenter, Reddi, & Anderson, 2009; Carpenter & Williams, 1995).

To facilitate comparisons between different data sets and conditions, the promptness data were quantified as cumulative probabilities on a probit scale (the inverse of the cumulative Gaussian distribution). In this so-called reciprobit format (Carpenter, 1988), a Gaussian distribution results in a straight line.

To investigate the promptness as a function of azimuth, the analysis was done within nine nonoverlapping, contiguous 15°-wide azimuth windows.

#### Statistical analysis

We estimated the mean across listeners and its 95% confidence interval for the following four parameters: gain γ, bias β, response variability σ, and promptness *P*. In what follows, we will denote the listening conditions (NH, 2CI, and 1CI), sound frequency bands (BB, HP, and LP), the azimuth bins (left, center, and right), for which the mean and confidence intervals had been estimated. We also indicate whether the mean and confidence interval are based on differences in those parameters (e.g., left–right, or 2CI-NH). Parameters were determined per listener on 5 to 20 responses. For the mean and confidence interval over these parameters, data were obtained from 11 (for NH and 2CI) and 7 listeners (for 1CI), respectively. We also denote the degrees of freedom (*df*) for every mean and confidence interval.

For graphical purposes, the standard error of the mean is shown in figures to indicate variability rather than the confidence interval.

## Results

### Horizontal Sound Localization

**Figure 4.**
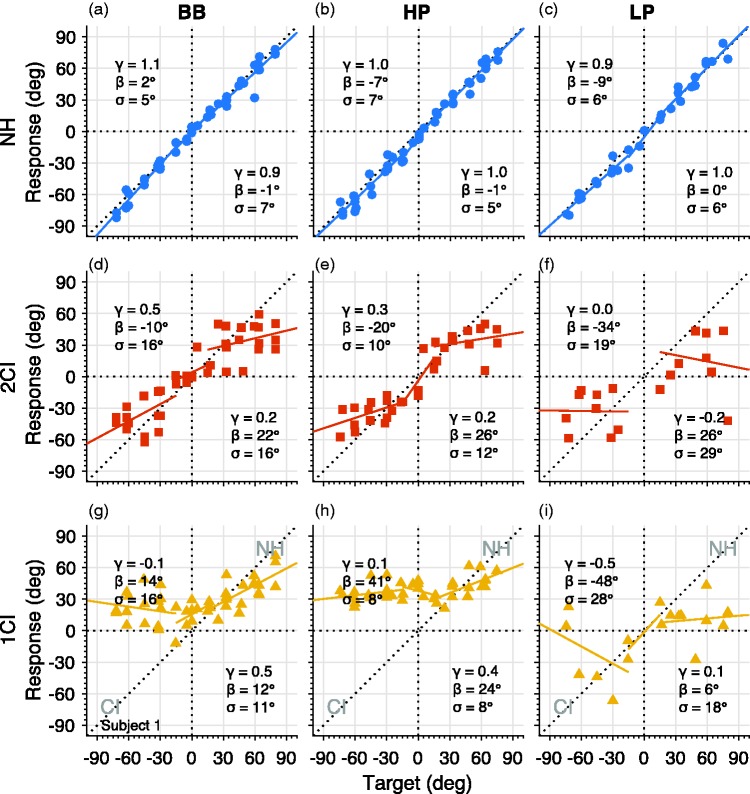
Azimuth stimulus–response plots for listener S1. (a–c) NH, (d–f) 2CI, and (g–i) 1CI hearing conditions. Gain (γ), bias (β), and response variability (σ) for the right (α > +15°) and left (α < −15°) sides are indicated in the lower-right and upper-left corners, respectively. BB = broadband; HP = high-pass; LP = low-pass; NH = normal hearing; 2CI = bilateral CI vocoder; 1CI: unilateral CI vocoder with a contralateral NH ear.

The use of a vocoder negatively affected sound localization performance in azimuth. Figure 4 illustrates the results from representative listener S1. With normal binaural hearing (Figure 4(a) to (c)), S1 localized BB, HP, and LP sounds in azimuth accurately and precisely in the free field. This near-perfect sound localization performance was expressed for all three azimuth ranges and all three sound types by an average gain of 1.0, a negligible response bias, and a small response variability of 6°.

When the sounds were real-time vocoded in both ears (Figure 4(d) to (f)), the listener had difficulty localizing sounds. The responses in the left and right bins barely depended on target location (mean gain = 0.2). Localization precision also decreased as evidenced by a larger scatter of the responses (response variability = 18°). The listener typically responded toward the sides with a pronounced response bias (negative for the left side [mean −23°], positive for the right side [mean +24°]).

Also, the central target locations could not be localized well by S1 (response variability ranged from 10° to 20°). Due to the limited number of targets (*N* = 1–7) and the large average response variability of 12°, it is hard to quantify the linear relationships for all three sound types in this central region. The central gain was low for BB sounds (0.4, Figure 4(d)), high for HP sounds (1.4, Figure 4(e)), and impossible to infer for the LP sounds (due to the low number of responses, Figure 4(f)).

In the 1CI condition, listener S1 performance was better on the nonvocoded side for these two sound types (mean gain on vocoded side = 0.0 and mean gain on nonvocoded side = 0.45). In contrast to BB and HP stimuli, the LP stimuli presented from the vocoded side were mostly detected toward the vocoder side with a mean bias of −21° (Figure 4(i)). The mean bias of 23° toward the NH (nonvocoded) ear for BB and HP sounds (Figure 4(g) and (h)). Moreover, the response variability was lowest for HP stimuli (8° on each side), higher for BB (16° and 11° for vocoded and nonvocoded side, respectively), and highest for LP sounds (28° and 18°, for vocoded and nonvocoded side, respectively).

**Figure 5.**
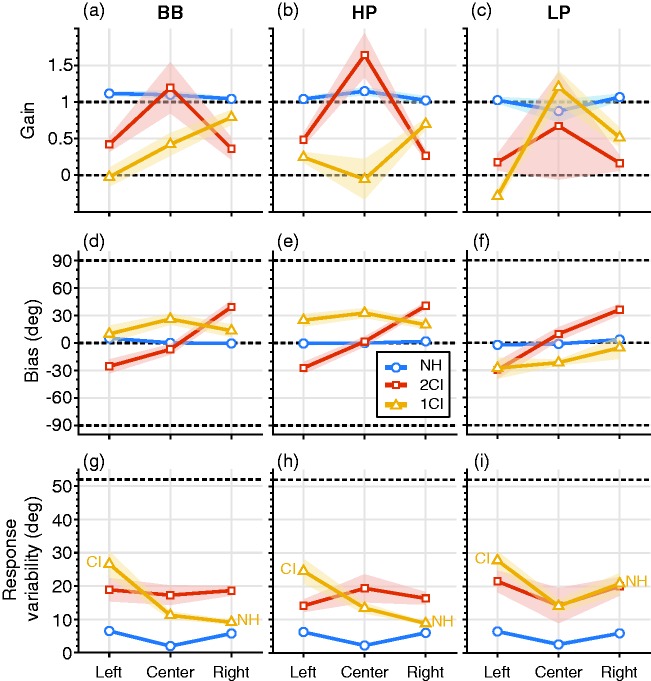
Azimuth localization performance. The regression parameter gains (top row), bias (center row), and variability (bottom row) are determined per azimuth bin (left, center, and right on the abscissa) for the three listening conditions (colored lines, normal hearing, NH; bilateral vocoder, 2CI; unilateral vocoder in one ear, 1CI) and the three sound frequency bands BB (left column), HP (middle column), and LP (right column). Open circles, colored patches denote mean and standard error across listeners, respectively. Dashed lines (a–c): perfect localization (γ = 1) and no localization (γ = 0) for the gains; (d–f): perfect localization (β = 0°) and complete left/right tendency (β = ±90°) for the biases; (g–i): response variability for perfect localization (σ = 0°) and for a completely random response behavior (σ ∼ 51°). BB = broadband; HP = high-pass; LP = low-pass.

An overview of the regression results (gain, bias and variability) for all listeners and for each listening condition and azimuth window is provided in Figure 5. The accurate localization performance of NH listening (Figure 5, blue) is evidenced by gains (γ) close to one, γ_(NH,_ *_df_*_ = 98)_ = 1 ± 0.05; Figure 5(a) to (c), response biases (β) near zero, β_(NH,_ *_df_*_ = 98)_ = 0.8 ± 2°; Figure 5(d) to (f), and a response variability (σ) of only a few degrees across the three azimuth windows, σ_(NH, _*_df_*_ = 98)_ = 5 ± 1.4°, for BB, HP, and LP stimuli (Figure 5(g) to (i)).

For 2CI listening conditions (Figure 5, red), all listeners showed strongly impaired response behavior. For BB and HP sounds, gains were small on both sides, γ_(2CI, right&left, BB&HP,_ *_df__ _*_= 43)_ = 0.4 ± 0.1, indicating that the systematic relation between target and response was substantially affected (Figure 5(a) to (c)). Together with a considerable bias on each side, |β|_(2CI, right&left, BB&HP,_ *_df_*_ = 43)_ = 34 ± 6°; Figure 5(d) to (f), listeners tended to respond at fixed left or right locations for 2CI listening.

In the 1CI listening condition (Figure 5, yellow), sound localization performance differed between the CI and NH side. Listeners localized sounds closer to normal on the NH side with higher accuracy and precision, γ_(1CI, NH-side, BB&HP,_ *_df_*_ = 13)_ = 0.7 ± 0.4; σ_(1CI, NH-side, BB&HP,_ *_df_*_ = 13)_ = 9 ± 1°, than on the vocoded side, γ_(1CI, CI-side, BB&HP,_ *_df_*_ = 13)_ = 0.1 ± 0.2, σ_(1CI, CI-side, BB&HP,_ *_df_*_ = 13) _= 26 ± 7°. Responses were biased toward the NH ear on both sides, β_(1CI, BB&HP,_ *_df_*_ = 41)_ = 26 ± 6°.

For LP sounds (Figure 5, right column), azimuth localization performance differed from localizing BB and HP sounds, both in the 2CI (Figure 5, red) and in the 1CI (Figure 5, yellow) listening conditions. In the 2CI condition, localization deteriorated, as the LP gain was slightly lower than for BB and HP stimuli, Δγ_(2CI, left&right, BB&HP-LP,_ *_df_*_ = 43)_ = 0.2 ± 0.1, and response variability was higher, Δσ_(2CI, left&right, BB&HP-LP,_ *_df__ _*_= 43)_ = −4 ± 3°. Biases did not differ, Δβ_(2CI, left&right, BB&HP-LP,_ *_df _*_= 43)_ = 3 ± 6°. In the 1CI condition, LP localization performance was also impoverished compared with BB and HP, even on the NH side. The gain decreased, Δγ_(1CI, NH-side, BB&HP-LP,_ *_df _*_= 13)_ = 0.23 ± 0.17, and response variability was higher, Δσ_(1CI, NH-side, BB&HP-LP,_ *_df_*_ = 13)_ = −11 ± 6°. While BB and HP localizations were biased toward the NH side, LP localization was biased toward the vocoder side, Δβ_(1CI, BB&HP-LP,_ *_df_*_ = 33)_ = 35 ± 10°.

To summarize, sound azimuth localization deteriorated for vocoded listening conditions. When both ears receive vocoded inputs, which perturbs ITDs and spectral cues, listeners tend to orient to fixed locations on their left and right. With only one ear receiving vocoded input, which leaves the monaural spectral cues on the NH side intact, localization performance changes in an azimuth-dependent way: for example, localization gains for HP and BB sounds increase from 0 on the left, vocoded side to about 0.7 on their right, NH side. In all vocoded listening conditions, however, LP localization, which for NH depends on ITD cues, was the worst.

### Vertical Sound Localization

**Figure 6.**
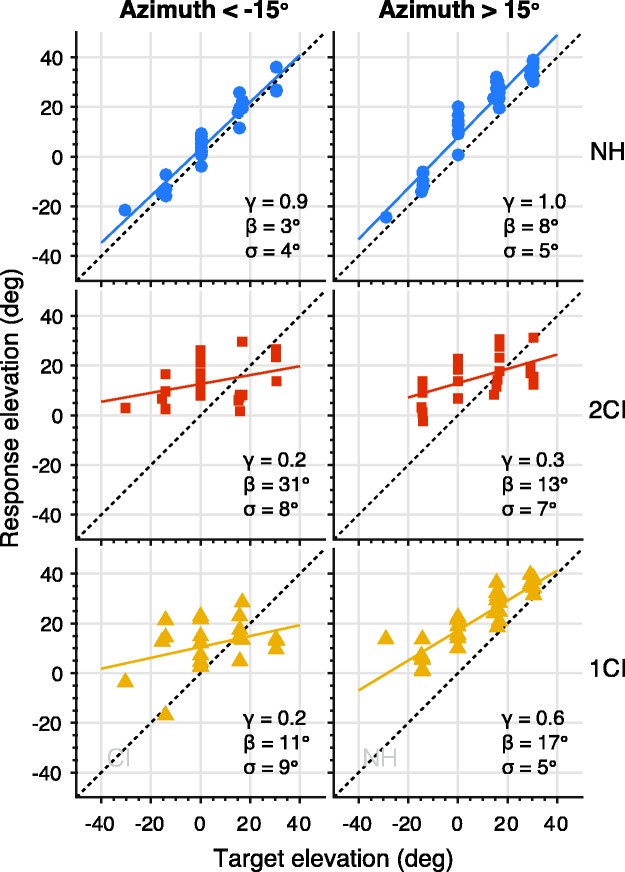
Elevation performance for listener S1 for BB and HP sounds combined. Gain (γ), bias (β), and response variability (σ) are presented for target azimuth α < −15° and α > 15° for NH (top, blue), 2CI hearing (center, red), and 1CI (bottom, yellow) conditions. The vocoder in the 1CI condition was on the left side (bottom left graph). NH = normal hearing; 2CI = bilateral CI vocoder; 1CI: unilateral CI vocoder with a contralateral NH ear.

As an illustrative example, Figure 6 shows the localization results in elevation of listener S1. In this case, the elevation responses are only shown for the left and right sides (<−15° and >+15°) to highlight that elevation localization performance can depend on sound source azimuth for certain conditions. For NH conditions, response elevation was accurate (mean gain = 1.0) and precise (mean response variability = 5°). In contrast, response elevation was virtually abolished for 2CI listening conditions on both hearing sides (mean gain = 0.25). For the 1CI listening condition, however, the elevation gain and response variability on the NH side (mean gain = 0.6; mean response variability = 5°) were substantially better than on the vocoded side (mean gain = 0.2; mean response variability = 9°), albeit lower than for NH.

**Figure 7.**
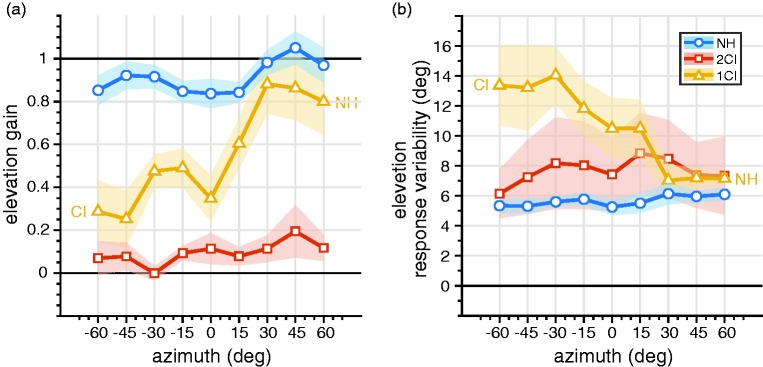
(a) Elevation gain and (b) response variability as a function of target azimuth. Open circles connected by lines denote the binned mean across listeners, whereas patches denote the standard error of the mean across listeners. Colors indicate NH, 2CI, and 1CI conditions. NH = normal hearing; 2CI = bilateral CI vocoder; 1CI: unilateral CI vocoder with a contralateral NH ear.

To quantify the behavior for all listeners, we used a regression analysis across narrower azimuth windows (see “Methods” section; Figure 7). For the NH listening condition, listeners localized sounds precisely in elevation (Figure 7(a), blue) across the entire azimuth range, γ_(NH,_ *_df _*_= 98)_ = 0.9 ± 0.1. In stark contrast, localization of elevation was nearly impossible for the 2CI listening condition (Figure 7(a), red) across the entire horizontal plane, γ_(2CI,_ *_df _*_= 98)_ = 0.1 ± 0.1.

Still, vertical sound localization behavior was also strongly affected by the vocoder in the 1CI condition, when the spectral pinna cues of only one ear were perturbed. While on the far contralateral (NH) side (azimuths ≥30°), elevation localization performance was only slightly below normal, γ_(1CI, ≥30°__,_ *_df _*_= 20)_ 0.8 ± 0.2, the gain decreased systematically toward the vocoded ear with a mean below 0.3, γ_(1CI, −60°__,_ *_df _*_= 6)_ = 0.3 ± 0.2. The effect of the unilateral vocoder on elevation performance was not only visible on the ipsilateral side, but it affected a substantial part of the NH side as well, γ_(1CI, 0&15°,_ *_df _*_= 13)_ = 0.5 ± 0.2.

The effects of listening condition and azimuth on elevation localization precision (Figure 7(b)) are similar to the effects on gain. With NH (Figure 7(b), blue), there is low variability (good performance) across the entire horizontal plane, σ_(NH,_ *_df _*_= 98)_ = 5.7° ± 1°. Response variability for the 2CI listening condition (Figure 7(b), red) is on average higher but also more idiosyncratic, σ_(2CI,_ *_df _*_= 98)_ = 7.7° ± 6°. Note that in this condition a low-response variability is still not indicative for a better localization performance, as the localization gain is near 0 (cf. Figure 7(a)). The response variability for 1CI hearing shows a gradual transition from high variability on the vocoder side, σ_(1CI, −60°,_ *_df _*_= 6)_ = 13° ± 6°, to near-normal variability on the far hearing side, σ_(1CI, 60_°_,_ *_df _*_= 6)_ = 7° ± 1°. Interestingly, the 1CI variability on the vocoder side is much higher than the 2CI variability (Figure 7(b)), while the ipsilateral 1CI gain is nearer the 2CI gain than the NH gain (Figure 7(a)).

### Promptness

**Figure 8.**
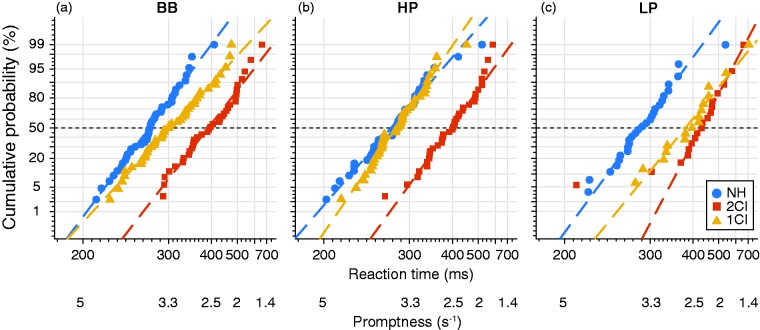
Promptness per spectral band (a–c) and listening condition (colors) for listener S1 on reciprobit scale. BB = broadband; HP = high-pass; LP = low-pass; NH = normal hearing; 2CI = bilateral CI vocoder; 1CI: unilateral CI vocoder with a contralateral NH ear.

Also, the response promptness was systematically affected by the acoustic manipulations of the vocoders. Figure 8 shows the promptness for listener S1 on reciprobit scale (see “Methods” section). In the NH listening condition (blue), the median value of this listener’s promptness for BB stimuli (found at a cumulative probability of 50%) was 3.6 ± 0.08 s^−1^ (Figure 8(a)). Bilateral vocoding of the sounds (red) substantially delayed the responses (lower mean promptness of 2.3 s^−1^). In the 1CI condition (yellow), the reaction times for BB sounds fell between the two conditions (mean promptness = 3.1 s^−1^). Similar reaction-time patterns were obtained for the HP (Figure 8(b)) and LP (Figure 8(c)) stimuli.

**Figure 9.**
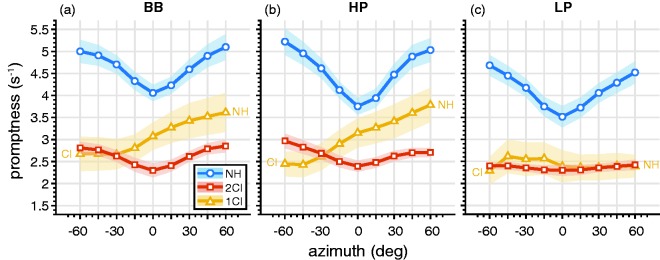
Promptness across azimuth for (a) BB, (b) HP, and (c) LP sounds. Open circles connected by lines denote binned mean promptness across listeners, whereas patches denote the standard error across listeners. Color indicates NH, 2CI, and 1CI conditions. BB = broadband; HP = high-pass; LP = low-pass; NH = normal hearing; 2CI = bilateral CI vocoder; 1CI: unilateral CI vocoder with a contralateral NH ear.

To quantify how promptness was affected by azimuth for the three hearing conditions for all listeners, we determined the mean promptness in narrow 20° azimuth windows (see “Methods” section; Figure 9). For NH listening (Figure 9, blue), responses were faster than for the other listening configurations across the entire azimuth range and for all three stimulus types, *P*_(NH,_ *_df _*_= 296)_ = 4.4 ± 0.1 s^−1^. The BB stimuli (Figure 9(a)) typically yielded the smallest promptness. In contrast, listeners responded slower for 2CI listening (red) regardless of stimulus location or spectral bandwidth, *P*_(2CI,_ *_df_*_ = 296)_ = 2.0 ± 0.05 s^−1^. In the 1CI listening condition, listeners responded faster on the NH side, *P*_(1CI, 60°__, BB-HP,_ *_df_*_ = 13)_ = 3.7 ± 0.6 s^−1^; Figure 9(a) and (b), and were systematically slower toward the CI side, *P*_(1CI, −60°__, BB-HP,_ *_df_*_ = 13)_ = 2.6 ± 0.5 s^−1^. For LP stimuli, listeners tended to respond equally fast across the entire azimuth range, *P*_(1CI, LP,_ *_df_*_ = 6)_ = 2.4 ± 0.7 s^−1^; Figure 9(c).

## Discussion

### Experimental Method

The experiments reported in this study are the first to have NH listeners using a real-time vocoder in free-field listening conditions. The use of natural head-orienting responses to assess sound localization performance allows for a fast, precise, and objective measurement of a listener’s localization abilities.

We used a target sound that was not a realistic, everyday sound. One might wonder why such a short, flat-spectrum stimulus was used. Nevertheless, such sounds are localized best by NH listeners (Blauert, 1996; Frens & Van Opstal, 1994; Goossens & Van Opstal, 1997, 1999; Hofman et al., 1998; Middlebrooks & Green, 1991) with highest accuracy and precision and fastest responses. In everyday environments, sounds of interest modulate in time and frequency over shorter and longer periods of time, move from one location to another (Hofman & Van Opstal, 2002; Van Barneveld & Van Wanrooij, 2013; Vliegen & Van Opstal, 2004), and are masked by other sounds from the background (Corneil, Van Wanrooij, Muñoz, & Van Opstal, 2002; Van Bentum et al., 2017; Van Wanrooij & Van Opstal, 2007) or from reflections (Brown et al., 2015; Ege, Van Opstal, Bremen, & Van Wanrooij, 2018; Litovsky, Colburn, Yost, & Guzman, 1999; Tollin & Yin, 2003). Identifying and localizing particular sounds in an auditory scene (Bregman, 1990) involve complex neural processing. Sound localization in these circumstances is difficult. It is hard to imagine that if localization of the simple stimuli in our study is impaired due to device processing that sounds from everyday dynamic environments would be easier to localize.

We measured several vocoder listening conditions in people with NH to investigate how device issues might affect spatial hearing. One aspect that we did not address was how the microphone positioning itself, without a specific speech processor, might affect sound localization cues, and hence performance. As such, our data should be seen as a result of a combination of device issues, including microphone position and processor properties.

### Device-Related Issues for Sound Localization

With veridical spatial cues present, NH listeners localize sound sources accurately (i.e., with small systematic errors) and precisely (i.e., with low variability) because of adequate binaural integration of the two monaural input streams.

Bilateral vocoders perturb the binaural and monaural localization cues considerably. The position of the BTE microphones affects two important factors: the head shadow decreases, which impacts the ILDs (Jones et al., 2016) and, because of its placement behind the pinnae, direction-dependent spectral-shape cues are absent. The reduced ILDs may restrict the azimuth response range, leading to deterioration in localization performance. ILDs may be further corrupted by CI automatic gain controls, including Oticon Medical’s instantaneous output compression. In our case, the instantaneous output compression knee point was set at 105 dB SPL and as the input levels were kept below this level both ears always had the same fixed input/output gain relationship (Figure 1(b)). In most CI systems, this would not be the case, and ILD cue corruption would occur due to automatic gain controls.

Furthermore, as current bilateral CI systems do not process temporal fine structure accordingly, fine structure ITDs are not accurately conveyed either. As a result, the vocoder devices create uncorrelated outputs across the ears (Figure 2(d)). In addition, the spectral resolution of the device is relatively poor, as typically only a few physical electrodes (or frequency bands) are operational (Loizou, 1998; Thakkar & Goupell, 2014; Figure 2(b) and (c)). This—together with the BTE microphone position—does not provide the necessary monaural spectral-shape cues needed for sound localization in the vertical plane.

Interaural coherence is also reduced for unilateral-vocoder listening because of the discordant inputs for the vocoder-stimulated and NH ear. As a result, the ILDs and ITDs are impoverished, making them inconsistent and poor localization cues. The low binaural coherence could lead to a reweighing of the localization cues (Agterberg, Hol, Van Wanrooij, Van Opstal, Snik, 2014; Van Wanrooij & Van Opstal, 2007). For example, if monaural cues (spectral cues and head-shadow cues) obtain a stronger weight for horizontal localization, performance on the NH side will yield a better target–response relationship than on the CI side. Similarly, monaural spectral-shape cues provide access to vertical localization on the NH side, but the contralateral vocoder with its poor spectral resolution may interfere with this capacity through binaural interactions in the central auditory system (Hofman & Van Opstal, 2002; Van Wanrooij & Van Opstal, 2005).

### Sound Localization With Bilateral Vocoders

Generally, our results show that acute listening with bilateral vocoders (2CI) immediately leads to a left-–right response pattern in NH listeners. This is in line with the results of horizontal localization with off-line vocoders (Jones et al., 2014). The access to accurate spatial hearing is highly perturbed, with listeners primarily reporting sounds coming either from the right or from the left (Figure 5), albeit with a nonzero gain on each side. This behavior might suggest that the perceived sound location is based on a judgment about which ear received the most intense sound. This localization strategy makes a comparison possible between two different signals but leads to an inaccurate response. Typically, similar response patterns have been reported for bilateral CI users (e.g., Grieco-Calub & Litovsky, 2010; Jones et al*.*, 2014; Kerber & Seeber, 2012).

Furthermore, vertical sound localization results in this listening condition is in line with earlier studies showing that complete removal of spectral pinna cues in both ears (e.g., Hofman et al., 1998; Van Wanrooij & Van Opstal, 2005) abolishes sound elevation localization in humans.

Together, these results suggest that perturbation of localization cues by the devices prevents CI users from accurately and precisely localizing sounds. Thus, we would argue that CI users with relatively unimpaired auditory pathways (e.g., little loss of spiral ganglion neurons) and without significant implant issues (e.g., little electrode insertion trauma) might have an improved sound localization performance if these device issues can be resolved. Other issues that were not examined in this study, such as electrode depth mismatch and spread of excitation (Francart & Wouters, 2007; Goupell, Stoelb, Kan, & Litovsky, 2013; Kan & Litovsky, 2015; Kan, Stoelb, Litovsky, & Goupell, 2013; Svirsky, Fitzgerald, Sagi, & Glassman, 2015), need to be addressed as well.

### Sound Localization With an NH Ear and a Single Vocoder

When listening with a vocoder and a contralateral NH ear, listeners localize sounds in the horizontal plane rather well on the nonvocoded side (Figure 5, yellow), suggesting that NH listeners can rely on monaural (spectral pinna) cues for sound localization in the horizontal plane (see also Van Wanrooij & Van Opstal, 2007). This percept depends on target azimuth, suggesting a weighting of spectral-shape cues along the horizontal plane. As direction-dependent spectral cues are not present in the LP stimuli, 1CI performance was generally worse in that particular frequency band for azimuth localization.

Also, elevation performance decreased (Figure 7, yellow). Previous studies have shown that accurate vertical sound localization is not only based on ipsilateral monaural cues but is also the result of a binaural integration process (Hofman & Van Opstal, 2003; Morimoto, 2001; Van Wanrooij & Van Opstal, 2005). The bilateral integration of spectral cues is affected as soon as one of the bilateral input is removed (or perturbed), and its effect compromises elevation performance on the nonperturbed side (Van Wanrooij & Van Opstal, 2005). Interestingly, the elevation response variability (Figure 7(b), yellow) seems to indicate that in the 1CI condition listeners tend to have an elevation percept on the vocoded side in each trial, although on average this percept is not accurate. To the authors’ knowledge, there are no studies on single-sided deaf (SSD) patients fitted with a CI that have quantified the effect of the device on vertical sound localization.

### Response Promptness

We argue that the difficulty of the listening task is systematically reflected in the promptness of the response. When binaural integration is accessible and accurate, a clear spatial percept can be rapidly constructed. Thus, the head-orienting responses to the targets can be fast, accurate, and precise. However, when the bilateral input is uncorrelated, and its binaural and spectral cues are perturbed, sound localization becomes more difficult, requiring alternative strategies to estimate the sound direction. When listeners listen through bilateral vocoders, reaction times increase (Figure 9, red), along with a deterioration in accuracy and precision (Figure 5, red). This effect is reduced when the auditory system exploits familiar acoustic cues, such as the monaural spectral cues for the 1CI condition. In this case, stimuli presented on the nonvocoded side elicit faster responses (Figure 9, yellow). Still, the NH side is affected by the lack of veridical binaural localization cues. The task (of responding as fast as possible) is harder than during bilateral NH, for which by far the shortest reaction times were obtained (Figure 9, blue).

Indeed, our results show that natural input (such as binaural fusion or monaural spectral cues) leads to fast reaction times and accurate and precise responses. In contrast, less-informative, confusing, or ambiguous listening conditions lead to longer reaction times and more localization errors. For example, LP stimuli elicit slower responses (Figure 9(c)), which could be attributed to the merely weak head shadow for low-frequency sounds and, therefore, increased difficulty in utilizing a bilateral loudness comparison strategy as observed for BB (Figure 9(a)) and HP (Figure 9(b)) sounds.

If the observed increment in reaction times reflects listening uncertainty, this might have implications for the use of a CI in the SSD. Although these people have slightly improved spatial hearing accuracy with their CI (Grossmann et al., 2016; Tavora-Vieir, De Ceulaer, Govaerts, & Rajan, 2015; Zeitler et al., 2015), the impact of CI use on sound localization reaction times is unknown. These people might have increased reaction times, similar to our acute 1CI condition. Despite clear CI benefits in speech perception and spatial hearing in simple, short-duration experiments, this aspect might actually reflect how much effort they need to invest for listening in everyday life situations over longer periods of time (Ohlenforst et al., 2017; Pichora-Fuller et al., 2016).

Note that lower promptness values (i.e., longer reaction times) were observed in the central azimuth region in both the NH and the 2CI condition. This has been reported in earlier studies (Frens & Van Opstal, 1995; Gabriel, Munoz, & Boehnke, 2010; Goldring, Dorris, Corneil, Ballantyne, & Munoz, 1996; Populin, 2008) and is referred to as the eccentricity effect. As this seems to be a consistent effect across several studies, it might be remarkable that it is not observed for the 1CI listening condition. We would pose that the eccentricity effect is not imposed by the physical target location but rather by the intended response movement. As such, an eccentricity effect is observed if promptness is binned on the basis of response eccentricity (not shown).

### Electrical Versus Acoustic Hearing

Electrical hearing in CI users differs fundamentally from acoustic hearing with a vocoder in NH listeners. For example, due to current spread and a potentially degraded auditory nerve, the effective number of frequency channels in a CI is typically less than 20. Furthermore, due to sensorineural hearing impairment (loss of outer hair cells), the dynamic range is severely impeded. Typically, an average electrical dynamic range is restricted to about 10 dB (Wouters, McDermott, & Francart, 2015) leading to a much more limited ILD range to be exploited.

In bilateral cochlear implantation, insertion asymmetries between electrodes can introduce strong perceptual differences across the ears (Svirsky et al., 2015). This produces even less-correlated bilateral inputs, leading to more difficulties in binaural integration than was observed in this study with bilateral, frequency-matched, vocoders (Goupell et al., 2013; Hu & Dietz, 2015; Kan et al., 2013). Uncorrelated bilateral input is even more pronounced for the SSD with a CI and one NH ear. This is why matching pitch and loudness (Veugen, Chalupper, Snik, Van Opstal, & Mens, 2016) may be very hard to achieve for bimodal CI users.

Most of these additional factors can in principle be simulated with the real-time vocoder. With this procedure, device-related issues and their impact on spatial hearing may be addressed with an NH auditory system, thus eliminating as much as possible highly variable, and often unknown, patient-related issues.

### Acute Versus Chronic Effects

In our study, we created acutely perturbed listening conditions, in which listeners were tested immediately after they were equipped with a 2CI or 1CI vocoder. Our data analysis indicates an acute reweighting of the available localization cues (e.g., as observed in the 1CI condition) as a strategy.

Although ILDs are the only possible veridical acoustic localization cue that can be exploited while hearing through the vocoder, and even though these cues were strongly perturbed as well, the learning brain might still be plastic enough to learn to use these cues, provided they are consistent and unique (Hofman et al., 1998; Kumpik, Kacelnik, & King, 2010; Van Wanrooij & Van Opstal, 2005). Thus, CI users might eventually learn to map these distorted cues to veridical source locations in the horizontal plane. In this study, we did not investigate the possibility of long-term learning, with potentially improved sound localization performance, through the vocoder. Nevertheless, without the availability of spectral cues and ITDs, which are congruent with the ILDs (as in natural sound fields), perceived source locations may always remain unresolved, for example, on the cone of confusion. Thus, we pose that spatial hearing for CI users might be feasible—not with binaural hearing based on ILD processing alone—but only if consistent, unambiguous spatial cue information is provided through the processors.

## Authors’ Note

Data are available from the Donders Institute for Brain, Cognition and Behaviour repository at https://data.donders.ru.nl and can be found via the following persistent identifier: http://hdl.handle.net/11633/aacdsm7e.
